# The Efficacy and Risk Profile of c-Met inhibitors in Non-small Cell Lung Cancer: a Meta-analysis

**DOI:** 10.1038/srep35770

**Published:** 2016-10-27

**Authors:** Sa Ye, Jiuke Li, Ke Hao, Jianping Yan, Hongbin Zhou

**Affiliations:** 1 Department of Respiratory Medicine, Zhejiang Provincial People’s Hospital, Hangzhou, Zhejiang, China; 2 Department of Ophthalmology, Sir Run Run Shaw Hospital, Zhejiang University School of Medicine, Hangzhou, China; 3 Department of Blood Transfusion, Zhejiang Provincial People’s Hospital, Hangzhou, Zhejiang, China

## Abstract

c-MET inhibitors are considered as a kind of novel drugs in non-small cell lung cancer (NSCLC) treatment. However, the results of different clinical studies involving c-MET inhibitors were not consistent. In this report, we performed Meta-analysis to investigate the beneficial and harmful effects of these drugs from 9 studies including 1611 patients in target drug groups and 1605 patients in control groups. As a result, patients in target drugs group had longer progression free survival (PFS) (HR 0.80, 95% CI 0.66–0.99, p = 0.04) but not overall survival (OS) than those in control group, especially in Asian (HR 0.57, 95% CI 0.42–0.76, p < 0.001), Non-squamous (HR 0.79, 95% CI 0.64–0.97, p = 0.03), Phase III (HR 0.66, 95% CI 0.50–0.86, p = 0.002), previous treated (HR 0.77, 95% CI 0.63–0.95, p = 0.01) and small molecular compounds subgroups (HR 0.62, 95% CI 0.50–0.78, p < 0.001). In addition, target drugs did not affect the objective response rate (ORR) but improved disease control rate (DCR) (RR 1.22, 95% CI 1.02–1.46, p = 0.03) of NSCLC patients. Our study first indicated that targeting c-MET therapies improved PFS and DCR in advanced or metastatic NSCLC patients, especially in previous treated Asian patients with adenocarcinoma.

As the leading cause of cancer-related death in the world, lung cancer is a major threat of health and heavy burden for family and society^1^,^2^. Traditionally, lung cancer is divided into small cell lung cancer (SCLC) and non-small cell lung cancer (NSCLC). The latter, accounting for nearly 80% of all lung cancer, can be further divided into squamous carcinoma, adenocarcinoma and large cell carcinoma by histology. However, this view should be renewed since the personalized medicine developed rapidly during the past decade^3^. It is of great importance to further classify NSCLC into specific subtypes with certain genetic markers, which is tightly related to therapeutic decision^3^. As the intrinsic trait of tumor cells, somatic mutation, chromosome rearrangement and copy number alterations existed in a large proportion of patients suffering from this disease^4^,^5^. Although the underlying mechanism of lung cancer has not been fully elucidated so far, it is widely accepted that some key genetic mutations in the airway epithelial cells play a pivotal role in the development of this malignancy.

There were many kinds of genomic aberrations observed in lung cancer patients, including epidermal growth factor receptor (EGFR) mutation and anaplastic lymphoma kinase (ALK) rearrangement, which are the most well known genetic alterations^6^,^7^. Comparatively, c-MET mutation is less common, and abnormal amplification of c-MET was found in about no more than 5% of NSCLC, mostly in adenocarcinoma^8^,^9^,^10^. Recent studies suggested that increased MET gene copy number or protein expression was conversely related to the prognosis of lung cancer, indicating a predictive value for this disease^11^,^12^. Subsequently, the drug inhibiting c-Met seems to be a new strategy for lung cancer management. In the past years, several kinds of drugs have been developed and applied into clinical trails, including tivantinib, crizotinib and onartuzumab etc. Nevertheless, the results of different clinical trails were not consistent^13^,^14^,^15^,^16^,^17^,^18^,^19^,^20^,^21^. For instance, the use of tivantinib prolonged the overall survival (OS) and progression-free survival (PFS) of patients with advanced lung cancer, while onartuzumab did not have an evident effect on PFS and OS during lung cancer therapy. The discrepancy might result from genetic background, different kinds of drugs and sample size. In order to determine the benefits and risks of the c-Met inhibitors, we conducted this meta-analysis to evaluate the efficacy and risk profiles of these drugs in lung cancer treatment.

## Results

### Characteristics of the included studies

**Figure 1:**
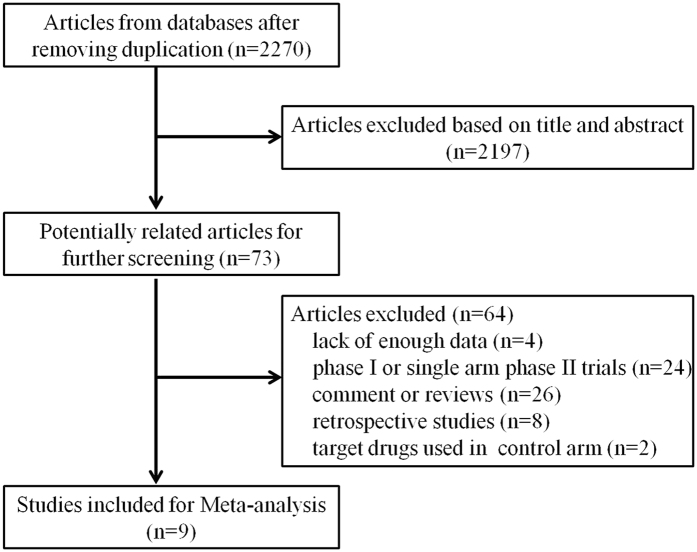
Study identification, inclusion, and exclusion criteria for the meta-analysis.

We identified 2270 relevant articles and abstracts, of which 73 studies were potentially suitable. 4 studies were eliminated due to lack of interest data, 24 were excluded because they were phase I or single-arm phase II trials, 26 were comments and reviews, 8 were retrospective studies and 2 studies with target drugs in both experimental and control arms. Thus, nine studies^13^,^14^,^15^,^16^,^17^,^18^,^19^,^20^,^21^, including 1611 patients from ten target drug groups and 1605 patients from ten control groups (the study by Wakelee *et al*. contained two experimental arms and two control arms), met the including criteria (Fig. 1). In addition, one article provided data of adverse events in several clinical trials about onartuzumab, which was not mentioned in other publications, so it was also included for our analysis^22^. Tivantinib and crizotinib were used in three and two studies, respectively, while onartuzumab was used as experimental drug in other four clinical trails. The study characteristics are listed in Table 1. All the patients were diagnosed by pathology and radiology. The quality of each study is listed in Table S1.

### Overall survival

8 trails reported overall survival (OS) data, however, no difference of hazard ratio (HR) were observed between experimental group and control group (HR 0.99, 95% CI 0.89–1.10, p = 0.87) (Tables 2 and S3). Moreover, subgroup analysis showed that target drugs did not prolong overall survival of NSCLC patients in almost every subgroup, even in high MET expression group (Table S2).

### Progression free survival

**Figure 2:**
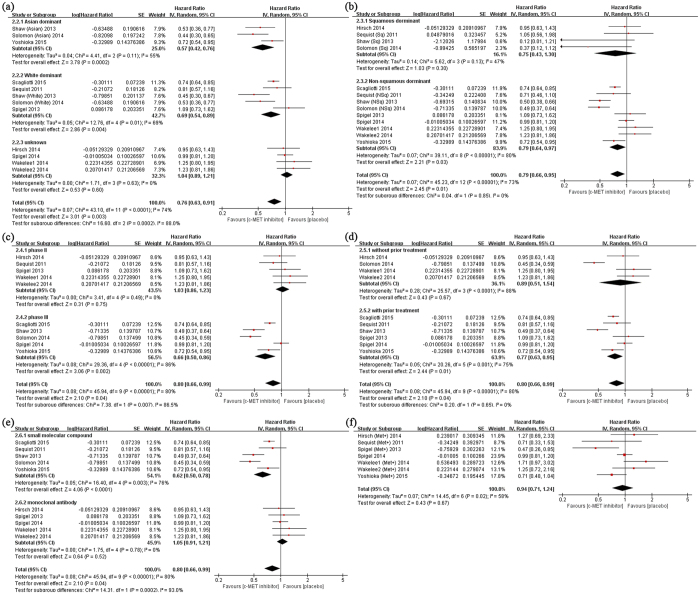
Subgroup analysis for progression-free survival (PFS) of NSCLC patients treated by c-MET inhibitors or placebo. The studies were divided into two or more subgroups according to (**a**) ethnicity (Asian dominant vs. White dominant vs. Unknown), (**b**) histology (Squamous dominant vs. Non-squamous dominant), (**c**) phase of clinical trails (Phase II vs. Phase III), (**d**) prior treatment (without prior treatment vs. with prior treatment), and (**e**) drug types (small molecular compounds vs. monoclonal antibody). In addition, high MET expression subgroup (**f**) was also applied to analysis.

All studies presented progression free survival (PFS) results, and the patients in target drugs group had longer PFS than those in control group (HR 0.80, 95% CI 0.66–0.99, p = 0.04) (Tables 2 and S3). Further analysis indicated that lengthened PFS in Asian dominant (HR 0.57, 95% CI 0.42–0.76, p < 0.001), Non-squamous (HR 0.79, 95% CI 0.64–0.97, p = 0.03), Phase III (HR 0.66, 95% CI 0.50–0.86, p = 0.002), second or later line treatment subgroups (HR 0.77, 95% CI 0.63–0.95, p = 0.01). While small molecular compounds inhibiting c-MET significantly prolonged PFS (HR 0.62, 95% CI 0.50–0.78, p < 0.001), there were no difference of PFS between monoclonal antibody (onartuzumab) treatment group and control group(HR 1.05, 95% CI 0.91–1.21, p = 0.52). Unexpectedly, we also failed to observe the beneficial effect of target drugs in high MET expression group (HR 0.94, 95% CI 0.71–1.24, p = 0.52) (Table S2 and Fig. 2).

### Objective response rate and disease control rate

**Figure 3:**
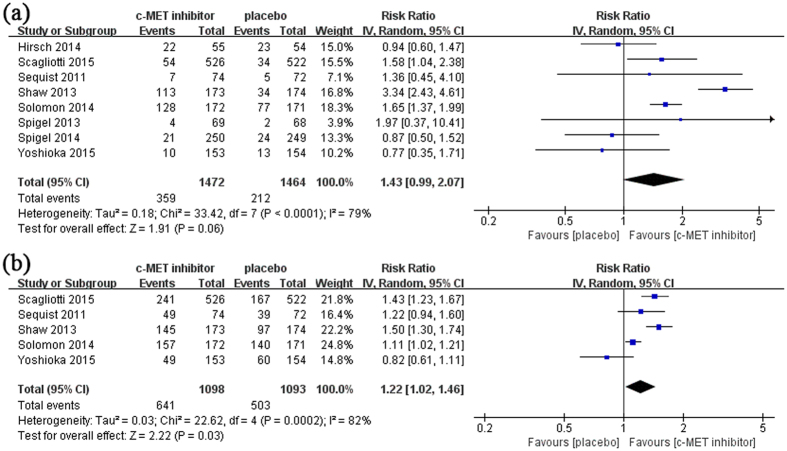
The effect of c-MET targeting therapy on objective response rate (ORR) and disease control rate (DCR). Overall ORR (**a**) and DCR (**b**) were calculated in available studies.

The information of objective response rate and disease control rate were available in 8 and 5 studies, respectively (Table 2). Target drugs did not affect the ORR (RR 1.43, 95% CI 0.99–2.07, p = 0.06) but improved DCR (RR 1.22, 95% CI 1.02–1.46, p = 0.03) of NSCLC patients (Table S3 and Fig. 3).

### Adverse events

Different kinds of adverse events were reported in each study, including anemia, neutropenia, rash, vomiting, diarrhea and so on. As a result, more patients with ILD (RR 4.47, 95% CI 1.15–17.45, p = 0.03, grade ≥ 3), edema (RR 3.23, 95% CI 2.24–4.64, p < 0.0001, all grades; RR 3.81, 95% CI 1.23–11.75, p = 0.02, grade ≥ 3) and respiratory infection (RR 2.24, 95% CI 1.63–3.07, p < 0.0001, all grades) was observed in target drug group. For other adverse events, there was no difference between experimental and control groups (Table 3).

### Sensitivity analysis

Sensitivity analysis was performed to observe the effect of a single study on the overall results. No study was found to affect the pooled OS value significantly. However, the effect of prolonged PFS disappeared if several studies were omitted. In addition, three studies by Hirsch *et al*., Spigel *et al*. and Yoshioka *et al*. had effect on the result of ORR^13^,^18^,^21^, because an improved ORR value with statistical significance would be obtained while withdrawing any of these studies. As for DCR, all the trials dramatically influenced this index except the one by Yoshioka *et al*.^21^ (Figure S1).

### Publication bias

**Figure 4:**
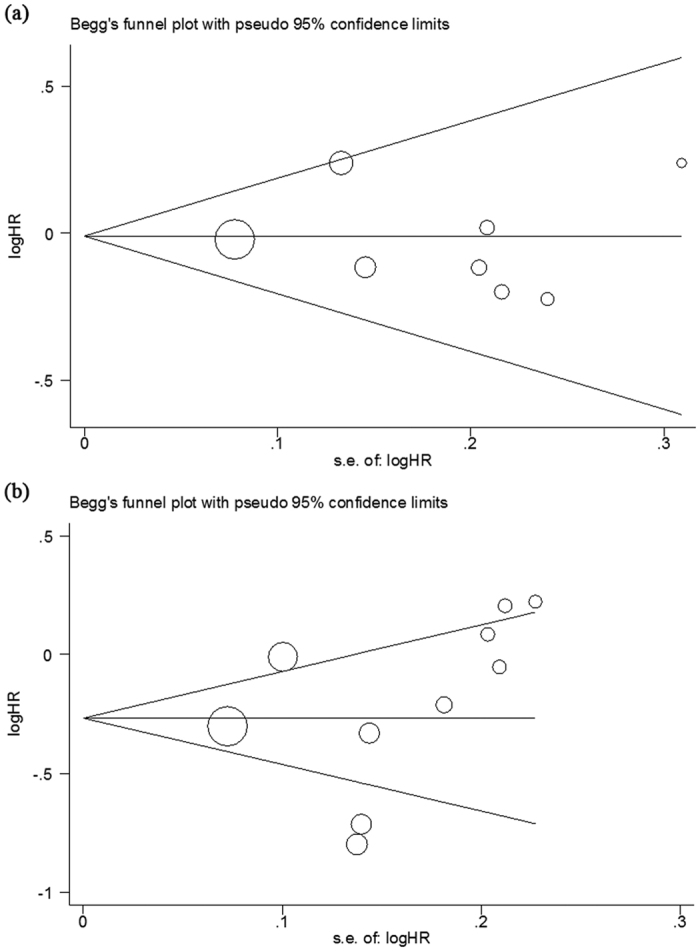
Publication bias on overall survival (OS) and progression free survival (PFS). (**a**) Begg’s funnel plot of the eligible studies involving OS; (**b**) Begg’s funnel plot of the eligible studies involving PFS.

Publication bias was tested using Begg’s and Egger’s tests. These tests did not show significant results in almost all comparisons (Table S3). The distribution of data points did not reveal evidence of obvious asymmetry (Fig. 4). These results indicated little publication bias.

## Discussion

Lung cancer is the most common malignant tumor globally, and the five-year survival rate is less than 5%^5^. The ideal treatment of lung cancer is radical operation. However, a large proportion of patients lost the opportunity of surgery, because the tumors are in advanced or metastatic stage once they get the affirmative diagnosis^23^. For advanced lung cancer, drug treatment is inevitable. Platinum-based chemotherapy has been applied in lung cancer treatment for a long time^24^. Despite chemotherapy played effective role in some patients, its limit is becoming more and more obvious in widely clinical appliance, such as drug resistance, adverse effects etc.^25^. There is an urgent need for novel drugs.

Genetic alteration is responsible for the acquirement of malignant characteristics, such as proliferation without limitation, resistance to apoptosis, metastasis, and etc.^26^. On the other hand, various kinds of mutations also provide an attractive strategy for lung cancer treatment. Since the discovery of EGFR mutation and the effect of Gefitinib, the first generation of small molecular tyrosine kinase inhibitor targeting EGFR pathway, much efforts has been made to seek out other molecular targets and develop novel drugs in the past years^27^,^28^. Some investigators aimed at c-MET due to its abnormal amplification in some NSCLC patients, and some kinds of agents have been applied to clinical trials and obtained contradictory results^7^,^9^,^29^. However, no comprehensive assessment of c-MET-targeting therapy in NSCLC treatment has been carried out so far.

In our study, we first found that the agents targeting c-MET significantly prolonged PFS but not OS in advanced or metastatic NSCLC patients, which was in consistence with the majority of individual studies. We assumed that several factors might lead to this result. First, all the enrolled patients were in stage IIIB or IV, which meant a large proportion of subjects had metastatic tumors in other organs. For these patients, the overall survival was limited. The target drugs were not powerful enough to prolong OS evidently. Second, for any individual study, the follow-up period can not cover the whole survival of all enrolled patients. The limited observational period might not find the difference of OS between experiment and control arms. Objective response rate (ORR) and disease control rate (DCR) are other indexes for evaluating the efficacy of drug in clinical trails. Our present data suggested that the patients treated by target drugs have a much higher DCR but not ORR than those with placebo treatment. According to definition, the disease control status included stable disease (SD), other than complete response (CR) and partial response (PR). Our results indicated that it was beneficial for patients with c-MET inhibiting agents treatment to maintain steady state, which was in line with the results that target drugs improved PFS. However, the reason why target drugs failed to improve OS and ORR was not clear.

In light of the great heterogeneity among clinical trials which might be associated with race, prior treatment, histology and drug types, subgroup analysis was performed to distinguish the confound factors. These factors seemed not affect the OS because no improvement of this index in any subgroup. However, PFS was much longer in Asian dominant, Non-squamaous, Phase III, with prior treatment subgroups, which provided a hint for selecting proper subpopulations in future clinical application. Compared with monoclonal antibody, only small molecular compounds (tivantinib and crizotinib) exhibited beneficial therapeutic effects in our analysis. The most possible reason was the manner in which drugs exerted their effects. Tivantinib is the first non-ATP competitive small molecular compound that targets the inactive kinase domain of MET^30^. The unique structure of the agent leads to the high selective action with MET^31^. Crizotinib, initially designed as c-MET inhibitor, is usually applied in patients with ALK arrangement due to its multi-target actions^32^. Onartuzumab, however, is a recombinant, humanized, monovalent, monoclonal antibody that binds with MET, thus preventing this receptor from combining with its ligand, hepatocyte growth factor (HGF)^33^. The different pharmacological mechanisms might led to the significant variance of therapeutic effect. Otherwise, the selection of proper patients and treatment period should also be taken into account.

The existence of obvious inconsistency suggested the potential of influence on overall results. As we expected, sensitivity analysis showed that all the studies had minor effect on OS, but several studies did have significant effect on PFS, ORR and DCR. Five studies were demonstrated to have influence on PFS, which was a positive result presented in this meta-analysis^14^,^15^,^16^,^17^,^21^. Among these, four studies with relatively greater weight reported positive effect favoring c-MET inhibitor^14^,^16^,^17^,^21^. Subsequently, this effect of PFS disappeared once removing one of these studies. Similar results were also observed when we analyzed DCR. In converse, excluding three studies would make the overall effect of ORR, a negative result in our work, favor c-MET inhibitor^13^,^18^,^21^. In view of the discrepancy among these studies, the therapeutic effect of c-MET inhibitor was not robust enough, and more well-designed studies would contribute to getting more reliable results.

The most surprising finding in our analysis was that no positive results were observed in high met expression subgroup. In light that these agents were targeting c-MET signal pathway, the patients with high met expression were naturally considered as the favorable population. As a matter of fact, only Spigel *et al*. found a longer PFS in high met expression subgroup in a phase II study evaluating onartuzumab^19^. It was just this result that promoted the start of a large-scale phase III clinical trial in which all the enrolled patients were met positive^18^. Disappointingly, the expected results did not appear and the trial was stopped forever. In spite of this, we tried to find out the possible reasons. First, the evaluation methods on c-MET expression were not consistent among these studies. For the majority of included trials, immunohistochemistry (IHC) was applied to detect c-MET protein expression^13^,^14^,^15^,^18^,^19^,^20^,^21^, while cMET amplification was detected by fluorescent *in situ* hybridization (FISH) in another trial by Sequist *et al*.^15^. As a result, the stratification based on c-MET expression is not unified, which may affect overall results. Second, we noticed that not all the subjects in these trials have clear information on c-MET expression or amplification. Subsequently, evaluating the effect of target drug become more difficult since a large part of subjects is lack of c-MET information. Third, it is worth to note that c-MET might also interact with other oncogenic signal pathways due to the existence of multi-variations in an individual patient. For example, both Engelman and Bean found MET amplification led to resistance to EGFR targeting therapy in EGFR mutant patients with adenocarcinoma, indicating the potential relationship between c-MET and EGFR pathway^34^,^35^. Thus, it is more reasonable to compare the effect of target drugs between high met expression group and low met expression group under similar EGFR status. Nevertheless, the information of both c-MET and EGFR mutation in an individual subject was hardly available, which prevented us from exploring the real effect of target drugs. Anyway, this unusual phenomenon still needs further investigation.

Any drug not only brings patients benefits, but also side effects. Various kinds of adverse events were reported in each study adapted to our research. These drugs might have harmful effect on haemopoietic, circulatory, respiratory, digestive and nervous system. While most adverse events occurred equally in experimental and control arms, more cases of ILD, edema and respiratory infection were found in patients with target drug treatment. In addition, the adverse events seemed to be drug specific. For example, all the ILD were found in tivantinib and crizotinib group and most edema cases were reported in patients with onartuzumab, which might be associated with the intrinsic characteristics of different drugs. In addition, genetic polymorphisms of metabolic enzymes existed in Asian patients, so the administration doses of tivantinib were different in ATTENTIONAL study according to patients’ genetic background^21^. However, a fixed administration dose was applied to every subject in other trials without considering the individual variation. The inappropriate dose of drugs might also be a potential factor to adverse events.

Our study had some shortages. First, some clinical trials were still ongoing and only a part of data was published in academic meeting. The incomplete data restricted us from further research. Second, as the genetic background is an important factor to the development of lung cancer, it should be well controlled in clinical studies. However, a part of subjects in several studies have no information of genetic alteration. It might lead to the confusing results. Third, there were at least four kinds of small molecular compounds inhibiting c-MET, but other drugs were not put forward to RCT, except tivantinib and crizotinib, which prevented us from carrying out an exhaustive assessment for all c-MET inhibitors. Moreover, while most patients recruited to clinical study were Asian and Whites, almost no data on Africans was available. The incomprehensive data might affect the reliability of this study.

As a summary, our study first indicated that targeting c-MET therapies improved PFS and DCR but not OS and ORR in advanced or metastatic NSCLC patients, especially in previous treated Asian patients with adenocarcinoma. Compared with tivantinib and crizotinib, onartuzumab did not display a beneficial effect for NSCLC patients. Despite some limitations, our study suggested that anti-c-MET pathway was a promising method for NSCLC treatment.

## Materials and Methods

### Search strategy

We performed a comprehensive search strategy in databases, including PubMed and Embase, to identify the researches about drugs targeting c-MET and lung cancer. The terms we used were as follows: “non-small cell lung cancer”, “NSCLC”, “c-met inhibitor”, “tivantinib”, “crizotinib”, “carbozantinib”, “foretinib”, “onartuzumab”. Additional studies were identified by a manual search from references of original studies or review articles on this topic. In order to ensure the integrity of the related studies, the relevant abstracts from academic meetings were also retrieved other than full text articles.

### Study Selection

The criteria for the selection of studies were as follows: (1) The studies should be randomized control trials (RCTs) with control arm and at least one experimental arm. (2) The drugs targeting c-MET in the studies should be used only in experimental arm. (3) The studies should provide at least one data of interest, such as overall survival (OS), progression free survival (PFS), objective response rate (ORR), disease control rate (DCR) and adverse effects (AE) etc. The studies in which the target drugs were used in both arms or the control arm was absent were excluded from our analysis.

### Data extraction

The data were independently extracted from all eligible publications by two authors according to the inclusion criteria listed above. Any disagreements were resolved by discussions with a third person. The information were extracted from the studies include the basic information of clinical trial (trial number, author, year and phase), the traits of enrolled patients (quantity, ethnicity, genetic background, histological types and prior treatment), the regimens in both arms, the clinical outcomes (OS, PFS), and the frequencies of ORR, DCR and AE in each arm.

### Quality assessment

All the enrolled trials were evaluated the quality with the help of the Cochrane five risk of bias domains tool, which included sequence generation, allocation concealment, data collection blinded, incomplete outcome data analyzing and selective outcome reporting. Each item was judged to obtain an assessment of “Yes” (low risk of bias), “No” (high risk of bias) or “Uncertain” (without enough information).

### Statistical Analysis

Hazard ratio (HR) and 95% confidence intervals (CIs) were used to assess the relative risk of OS and PFS between experimental arm and control arm in the studies. While analyzing ORR, DCR and AE in target drug groups and control groups, relative risk (RR) and 95% confidence intervals (CIs) were applied.

I^2^ statistic was used to quantify the degree of heterogeneity, with I^2^ <25%, 25–75% and >75% representing low, moderate and high degrees of inconsistency, respectively^36^,^37^. In the analysis of pooled data, we used two different models according to the traits of the included studies: If no heterogeneity was found, a fixed effect model was adopted to determine the effects of target drugs or a random effect model was applied. If heterogeneity existed across studies, a subgroup analysis was performed to seek out the source of heterogeneity. The studies were subdivided by ethnicity (Asian dominant vs. White dominant), histological types (Squamous vs. Non-Squamous), prior treatment (first-line vs. second or later line), clinical trial phase (Phase II vs. Phase III) and different drug types (small molecular compounds vs. monoclonal antibody). Moreover, the patients with high MET expression were listed as a single subgroup. Sensitivity analysis was also performed to check the stability of the overall effect.

We made use of a Begg’s funnel plot to examine the underlying publication bias and Egger’s weighted regression method to calculate a P value for bias^38^,^39^. If no publication bias exists, the funnel plot appears symmetrical.

All analyses were conducted with the use of Review Manager, V.5.2 (Revman, The Cochrane Collaboration, London, UK) or STATA software, V.12.0 (StataCorp LP, College Station, TX, USA).

## Additional Information

**How to cite this article**: Ye, S. *et al*. The Efficacy and Risk Profile of c-Met inhibitors in Non-small Cell Lung Cancer: a Meta-analysis. *Sci. Rep.* **6**, 35770; doi: 10.1038/srep35770 (2016).

**Publisher’s note:** Springer Nature remains neutral with regard to jurisdictional claims in published maps and institutional affiliations.

## Supplementary Material

Supplementary Information

Supplementary Information 1

## Figures and Tables

**Table 1:** Information of enrolled randomized control trials.

Year	Author	NCT	Phase	Race	Histology	Tumor stage	Prior therapy	Primary end point	Secondary end point
2014	Hirsch	01519804	II	unknown	Squamous	IIIB/IV	No	PFS	OS/ORR/safety
2015	Scagliotti	01244191	III	mixed, White dominant	Adeno- carcinoma dominant	IIIB/IV	Yes	OS	PFS/safety
2011	Sequist	00777309	II	mixed, White dominant	Non-squamous dominant(70%)	IIIB/IV	Yes	PFS	OS/ORR/safety
2013	Shaw	00932893	III	mixed, Asian (46%). Non- Asian (54%)	Adeno- carcinoma dominant	IIIB/IV	Yes	PFS	OS/ORR/safety
2014	Solomen	01154140	III	mixed, Asian (46%), Non- Asian (54%)	Adeno- carcinoma dominant	IIIB/IV	No	PFS	OS/ORR/safety
2013	Spigel	00854308	II	mixed, White dominant	Non-squamous dominant(71%)	IIIB/IV	Yes	PFS	OS/ORR/safety
2014	Spigel	01456325	III	unknown	Adeno- carcinoma dominant	IIIB/IV	Yes	OS	PFS/ORR/safety
2014	Wakelee1	01496742	II	unknown	Non-squamous	IIIB/IV	No	PFS	OS/ORR/safety
2014	Wakelee2	01496742	II	unknown	Non-squamous	IIIB/IV	No	PFS	OS/ORR/safety
2015	Yoshioka	01377376	III	Asian	Non-squamous	III-IV	Yes	OS	PFS/ORR/DCR/safety
**Year**	**Author**	**c-MET detection**	**Control arm**			**Experimental arm**			
			**regimen**	**number**	**c-MET+/−**	**regimen**	**number**	**c-MET+/−**	
2014	Hirsch	IHC	Pac + Plat + Pb^*^	54	NA	Pac + Plat + Onartuzumab	55	NA	
2015	Scagliotti	IHC	Erl + Pb^#^	522	107/127	Erl + Tivantinib	526	104/107	
2011	Sequist	FISH	Erl + Pb^#^	83	18/35	Erl + Tivantinib	84	19/38	
2013	Shaw	NA	Pem or Doc^◊^	174	NA	Crizotinib	173	NA	
2014	Solomen	NA	Pem or Plat^◊◊^	171	NA	Crizotinib	172	NA	
2013	Spigel	IHC	Erl + Pb^**^	68	31/31	Erl + Onartuzumab	69	35/31	
2014	Spigel	IHC	Erl + Pb^**^	249	249/0	Erl + Onartuzumab	250	250/0	
2014	Wakelee1	IHC	Pac + Plat + Bev + Pb^***^	70	NA	Pac + Plat + Bev + Onartuzumab	69	NA	
2014	Wakelee2	IHC	Pem + Plat + Pb^****^	61	NA	Pem + Plat + Onartuzumab	59	NA	
2015	Yoshioka	IHC	Erl + Pb^#^	153	77/69	Erl + Tivantinib^##^	154	83/65	

Abbreviation: Bev - Bevacizumab; Doc - Docetaxel; Erl - Erlotinib; Pac - Paclitaxel; Pb - Placebo; Pem - Pemetrexed; Plat - Carboplatin or Cisplatin; NA: Not available. *Patients receive four 3-weekly cycles of paclitaxel (200 mg/m2 i.v. d1) with carboplatin or cisplatin (investigators’ choice; standard doses) plus either Onartuzumab (15 mg/kg i.v. d1) or Pb. **Onartuzumab (15 mg/kg diluted in 0.9% normal saline solution to total volume of 250 cm3) or placebo (250 cm3 0.9% normal saline solution provided by investigative site) was administered by intravenous infusion every 3 weeks. Erlotinib was administered orally at 150 mg daily. ***Patients receive either Onartuzumab (15 mg/kg i.v. q3w) or Pb combined with paclitaxel/platinum/bevazumab (Pac/Plat/Bev) 4 cycles. ****Patients receive either Onartuzumab (15 mg/kg i.v. q3w) or Pb in combination with Plat/Pem 4 cycles. ^#^Patients receive oral erlotinib (150 mg daily) plus oral tivantinib (360 mg twice daily) or erlotinib plus placebo. ^##^The dose of tivantinib was based on pretreatment testing for CYP2C19 genotype: Extensive metabolizers (Ems) received 360 mg b.i.d. and poor metabolizers (PMs) received 240 mg b.i.d. ^◊^Patients receive oral crizotinib (250 mg twice daily) in a 3-week cycle or intravenous chemotherapy comprising either pemetrexed (500 mg per square meter of body-surface area) or docetaxel (75 mg per square meter) every 3 weeks. ^◊◊^Patients receive oral crizotinib, at a dose of 250 mg twice daily, or intravenous chemotherapy (pemetrexed, at a dose of 500 mg per square meter of body-surface area, plus either cisplatin, at a dose of 75 mg per square meter, or carboplatin, target area under the curve of 5 to 6 mg per milliliter per minute) administered every 3 weeks for a maximum of six cycles.

**Table 2:** The efficacy of c-Met inhibitor in advanced or metastatic NSCLC treatment.

Year	Author	OS			PFS			ORR		DCR	
		Target arm (months)*	Control arm (months)*	HR [95% CI]	Target arm (months)*	Control arm (months)*	HR [95% CI]	Target arm**	Control arm**	Target arm***	Control arm***
2014	Hirsch	NA	NA	1.27 [0.69–2.32]	4.9	4.9	0.95 [0.63–1.43]	22/55	23/54	NA	NA
2015	Scagliotti	8.5 [7.1–9.3]	7.8 [7.0–9.0]	0.98 [0.84–1.14]	3.6 [2.8–3.7]	1.9 [1.9–2.0]	0.74 [0.64–0.85]	54/526	34/522	241/526	167/522
2011	Sequist	8.6 [7.0–10.3]	6.9 [5.6–10.4]	0.87 [0.59–1.27]	3.8 [3.2–5.4]	3.3 [1.9–3.7]	0.81 [0.57–1.16]	7/74	5/72	49/74	39/72
2013	Shaw	20.3 [18.1-NR]	22.3 [18.6-NR]	1.02 [0.68–1.54]	7.7 [6.0–8.8]	3.0 [2.6–4.3]	0.49 [0.37–0.64]	113/173	34/174	145/173	97/174
2014	Solomen	NA	NA	0.82 [0.54–1.26]	10.9 [8.3–13.9]	7.0 [6.8–8.2]	0.45 [0.35–0.60]	128/172	77/171	157/172	140/171
2013	Spigel	8.9	7.4	0.80 [0.50–1.28]	2.2	2.6	1.09 [0.73–1.62]	4/69	2/68	NA	NA
2014	Spigel	6.8 [6.1–7.5]	9.1 [7.7–10.2]	1.27 [0.98–1.65]	2.7	2.6	0.99 [0.81–1.20]	21/250	24/249	NA	NA
2014	Wakelee1	NA	NA	NA	NA	NA	1.25 [0.80–1.95]	NA	NA	NA	NA
2014	Wakelee2	NA	NA	NA	NA	NA	1.23 [0.81–1.86]	NA	NA	NA	NA
2015	Yoshioka	12.7 [10.0–15.7]	11.1 [9.5–12.6]	0.89 [0.67–1.18]	2.9 [2.0–3.8]	2.0 [1.2–3.4]	0.72 [0.54–0.95]	10/153	13/154	49/153	60/154

Abbreviations: NA - Not available; NR - Not reached; OS - Overall survival; PFS - Progression-free survival; ORR - Objective response rate; DCR - Disease control rate; HR - Hazard ratio. *The data were presented as median [95% CI] if 95% CI was available. If not, only median was listed. **The data were presented as subjects with objective response/total subjects in an arm. ***The data were presented as subjects with disease control/total subjects in an arm.

**Table 3:** The risk profiles of c-Met inhibitor in advanced or metastatic NSCLC treatment.

Adverse events	All Grades			Grade ≥ 3		
	No. of trials	RR [95% CI]	P value	No. of trials	RR [95% CI]	P value
Abnormal liver function	4	1.68 [0.82, 3.46]	0.16	3	2.67 [0.54, 13.21]	0.23
Anemia	5	1.16 [0.47, 2.85]	0.75	6	1.03 [0.26, 4.11]	0.96
Constipation	4	1.42 [1.00, 2.03]	0.05	2	7.88 [0.99, 62.79]	0.05
Cough	3	1.17 [0.96, 1.44]	0.13	1	1.49 [0.42, 5.25]	0.53
Dermatitis acneiform	3	1.00 [0.68, 1.49]	0.98	2	1.39 [0.44, 4.37]	0.57
Diahhrea	6	1.36 [0.82, 2.26]	0.23	5	0.90 [0.54, 1.52]	0.70
Dyspnea	5	0.96 [0.82, 1.12]	0.60	5	0.94 [0.60, 1.47]	0.77
Edema	7	**3.23 [2.24, 4.64]**	**<0.0001**	6	**3.81 [1.23, 11.75]**	**0.02**
Fatigue	5	0.91 [0.74, 1.12]	0.37	5	1.10 [0.78, 1.56]	0.57
ILD	3	1.37 [0.55, 3.45]	0.5	2	**4.47 [1.15, 17.45]**	**0.03**
Leukopenia	3	3.64 [0.19, 69.27]	0.39	2	3.70 [0.02, 568.74]	0.61
Nausea	5	1.07 [0.88, 1.30]	0.49	5	0.50 [0.22, 1.11]	0.09
Neutropenia	4	3.70 [0.73, 18.66]	0.11	5	2.55 [0.78, 8.40]	0.12
Pyrexia	3	1.15 [0.50, 2.64]	0.74	3	0.55 [0.09, 3.37]	0.52
Rash	6	0.96 [0.84, 1.10]	0.53	3	0.95 [0.46, 1.97]	0.90
Respiratory infection	3	**2.24 [1.63**, **3.07]**	**<0.0001**	3	1.24 [0.34, 4.60]	0.74
Vomiting	5	1.25 [0.76, 2.05]	0.39	4	1.00 [0.44, 2.24]	0.99
VTE	4	1.80 [0.87, 3.70]	0.11	4	1.78 [0.88, 3.59]	0.11

Abbreviations: ILD - Interstitial lung disease; VTE - Venous thromboembolism.
