# Supplementary material for: The Efficacy and Risk Profile of c-Met inhibitors in Non-small Cell Lung Cancer: a Meta-analysis
Source: Sci Rep. 2016 Oct 27;6:35770. doi: 10.1038/srep35770 (PMC5081544; doi:10.1038/srep35770)
Supplement: Supplementary Information [file srep35770-s1.doc]

**The Efficacy and Risk Profile of c-Met inhibitors in Non-small Cell Lung Cancer: a Meta-analysis**

Sa Ye^1^, Jiuke Li^3^, Ke Hao^2^, Jianping Yan^1^, Hongbin Zhou^1*^

^1^Department of Respiratory Medicine, Zhejiang Provincial People’s Hospital, Hangzhou, Zhejiang, China

^2^Department of Blood Transfusion, Zhejiang Provincial People’s Hospital, Hangzhou, Zhejiang, China

^3^Department of Ophthalmology, Sir Run Run Shaw Hospital, Zhejiang University School of Medicine, Hangzhou, China.

* Corresponding author: Hongbin Zhou, Department of Respiratory Medicine, Zhejiang Provincial People’s Hospital, 158 Shangtang Road, Hangzhou, 310014, China; Email: zhb0401@126.com

**Sensitivity analysis of each study on the therapeutic effects of c-MET inhibitors**


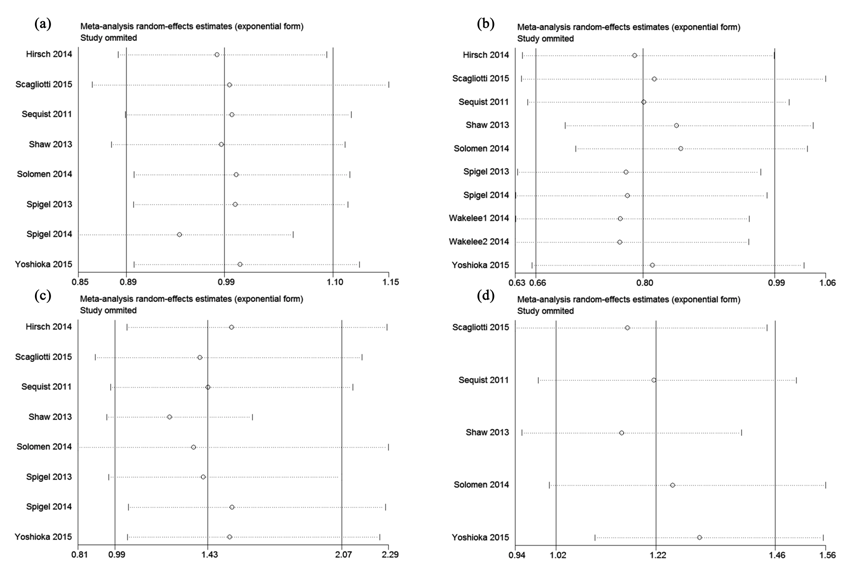


**Figure S1** the influence of each study on the overall therapeutic effect of c-MET inhibitor treatment. (a) Overall survival (OS); (b) progression-free survival (PFS); (c) objective response rate (ORR); (d) disease control rate (DCR)

Table S1 Quality assessment of enrolled clinical trials

| Year | Author | Sequence generation | Allocation concealment | Data collection blinded | Incomplete outcome data analysis | Selective outcome reporting |
| --- | --- | --- | --- | --- | --- | --- |
| 2014 | Hirsch | No | Uncertain | Yes | Yes | Yes |
| 2015 | Scagliotti | Yes | Yes | Yes | Yes | Yes |
| 2011 | Sequist | Yes | No | Yes | Yes | Yes |
| 2013 | Shaw | Yes | Uncertain | Yes | Yes | Yes |
| 2014 | Solomen | Yes | Uncertain | Yes | Yes | Yes |
| 2013 | Spigel | No | Yes | Yes | Yes | Yes |
| 2014 | Spigel | Yes | Yes | Yes | Yes | Yes |
| 2014 | Wakelee | No | Uncertain | Yes | Yes | Yes |
| 2015 | Yoshioka | Yes | Yes | Yes | Yes | Yes |

TableS2 Subgroup analysis of pooled results for OS and PFS in enrolled clinical trials

| Subgroup | OS | | | |  | PFS | | | |
| --- | --- | --- | --- | --- | --- | --- | --- | --- | --- |
|  | No. of trials | HR [95%CI] | P | I2 |  | No. of trials | HR [95%CI] | P | I2 |
| **Ethnicity** |  |  |  |  |  |  |  |  |  |
| Asian dominant | 1 | 0.89 [0.67, 1.18] | 0.43 | NA |  | 3 | 0.57 [0.42, 0.76] | 0.0002 | 55% |
| White dominant | 3 | 0.95 [0.83, 1.09] | 0.49 | 0 |  | 5 | 0.69 [0.54, 0.89] | 0.004 | 69% |
| Mixed* | 2 | 0.92 [0.68, 1.23] | 0.57 | 0 |  | NA | NA | NA | NA |
| unknown | 2 | 1.27 [1.00, 1.61] | 0.05 | 0 |  | 4 | 1.04 [0.89, 1.21] | 0.6 | 0 |
| **Histology** |  |  |  |  |  |  |  |  |  |
| Squamous dominant | 2 | 0.94 [0.57, 1.55] | 0.81 | 0 |  | 4 | 0.75 [0.43, 1.30] | 0.3 | 47% |
| Non-squamous dominant | 7 | 0.99 [0.89, 1.10] | 0.8 | 0 |  | 9 | 0.79 [0.64, 0.97] | 0.03 | 80% |
| **Phase** |  |  |  |  |  |  |  |  |  |
| phase II | 3 | 0.92 [0.70, 1.21] | 0.56 | 0 |  | 5 | 1.03 [0.86, 1.23] | 0.75 | 0 |
| phase III | 5 | 1.00 [0.88, 1.14] | 0.95 | 16% |  | 5 | 0.66 [0.50, 0.86] | 0.002 | 86% |
| **Previous treatment** |  |  |  |  |  |  |  |  |  |
| No | 2 | 0.96 [0.64, 1.46] | 0.87 | 26% |  | 4 | 0.89 [0.51, 1.54] | 0.67 | 88% |
| Yes | 6 | 1.00 [0.89, 1.11] | 0.94 | 2% |  | 6 | 0.77 [0.63, 0.95] | 0.01 | 75% |
| **Drug** |  |  |  |  |  |  |  |  |  |
| small molecular compound | 5 | 0.95 [0.84, 1.06] | 0.36 | 0% |  | 5 | 0.62 [0.50, 0.78] | <0.0001 | 76% |
| monoclonal antibody | 3 | 1.12 [0.84, 1.50] | 0.45 | 32% |  | 5 | 1.05 [0.91, 1.21] | 0.52 | 0% |
| **Genetic background** | |  |  |  |  |  |  |  |  |
| MET positive | 6 | 0.79 [0.56, 1.10] | 0.17 | 68% |  | 7 | 0.94 [0.71, 1.24] | 0.67 | 58% |

Abbreviations: NA: Not available; OS: Overall survival; PFS: Progression-free survival; HR: Hazard ratio

* Asians occupied about a half of total subjects in two trials reported by Shaw et al and Solomon et al, and the remain patients were mostly Whites. The data about OS for each race were not available, while the data on PFS for each race were listed in these trials.

Table S3 Pooled HR or RR for efficacy of c-MET inhibiting therapy, heterogeneity and publication bias in meta-analysis: comparison between target arm and control arm

| Index | No. of trials | HR/RR [95% CI]* | P value | Heterogeneity | | Publication bias | |
| --- | --- | --- | --- | --- | --- | --- | --- |
|  |  |  |  | I^2^ | P _heterogeneity_ | Begg | Egger |
| OS | 8 | 0.99 [0.89, 1.10] | 0.87 | 0 | 0.48 | 0.536 | 0.773 |
| PFS | 10 | 0.80 [0.66, 0.99] | 0.04 | 80% | <0.0001 | 0.074 | 0.48 |
| ORR | 8 | 1.43 [0.99, 2.07] | 0.06 | 79% | <0.0001 | 0.902 | 0.406 |
| DCR | 5 | 1.22 [1.02, 1.46] | 0.03 | 82% | 0.0002 | 0.806 | 0.903 |

* For OS and PFS, the data were showed as HR [95%CI]; For ORR and DCR, data were showed as RR [95%CI]
